# Supplementary material for: Effects of early life adversity on immediate early gene expression: Systematic review and 3-level meta-analysis of rodent studies
Source: PLoS One. 2022 Jan 13;17(1):e0253406. doi: 10.1371/journal.pone.0253406 (PMC8757918; doi:10.1371/journal.pone.0253406)
Supplement: Attachment — Submitted filename: mIEG_plos_reviewers_MJ.docx [file pone.0253406.s003.docx]

**Answers to reviewers’ comments**

**Reviewer #1:** Schuler et al. reported effects of early life adversity (ELA) on IEG expression in mice and rats by using a systemic review and meta-analysis. This is an interesting and valuable paper giving a general overview on the relationship between ELA and IEG expression and highlighting current knowledge gaps. There are, however, several issues to be addressed to further improve the manuscript.

1. The authors carefully performed a systemic literature search by listing strict inclusion and exclusion criteria described in the protocol (S1.1). However, no matter how careful and sufficient criteria are set, it would be undeniable that judgment is suffered from subjectivity. Scientific reasons to exclude 2192 publications shown in Fig.2, particularly, wrong outcome, wrong intervention, would be unclear.

We agree that the terminology “wrong” may mislead the reader. We appropriately removed the word “wrong” and specified in the figure caption that studies were not included if they did not report outcomes / population / intervention that matched our prespecified inclusion/exclusion criteria.

2. As the authors mentioned in the Discussion part, the authors restricted their meta-analysis to adult animals only, and they speculated different patterns of IEG expression associated to ELA exposure during different developmental stages. Developing and juvenile stages in rodent brain are critical to neuronal development and HPA-axis programing. Thus, the comparison between these stages and adult stage should be important to understand effects of ELA on IEG.

We agree with the reviewer that investigating the juvenile period is essential for critical neuronal development and HPA axis programming. Investigating the effects of ELA on IEG expression through developmental stages was outside the scope of this review, and it would be better investigated with an experimental (rather than meta-analytical) design. We have included a sentence in the discussion to underline the importance of future longitudinal experimental studies (**lines 390-395**):

*“Lastly, given the expected heterogeneity in study designs, we restricted our meta-analysis to adult animals only, and – at this stage – it cannot be generalized to other age groups. It is possible that different patterns of IEG expression associated to ELA exposure may emerge in juvenile or adolescent animals. Future experiments investigating the longitudinal effects of ELA on IEG expression over the course of development can shed light on the interaction between ELA, development and IEG-related brain activity.”*

3. In the supplemental section, the authors mentioned “study inclusion and risk of bias are assessed by two independent researchers, and discrepancies will be resolved by discussion between these two experimenters. Should no conclusion be reached between two experimenters, a third researcher (expert in the field of early life adversity), will be consulted for a solution.” Please clearly show the criteria of expert in the field of early life adversity. Because the role of expert should be critical to the present study.

The initials of the expert (MJ) have been added to the text. Although no definition of “expert” was formally defined prior to the project, MJ has an outstanding scientific track record in this field (https://scholar.google.com/citations?user=djZ9vCQAAAAJ&hl=nl).

**Reviewer #2: manuscript PONE-D-21-18961**

In this study Schuler and Colleagues investigate, by systematic reviewing and meta-analyzing the literature, the long-term effects of early life adversities (ELA) on immediate early genes (IEG) brain expression. The authors select as ELA the experimental manipulations affecting maternal cares carried out during the three first post-natal weeks and results report IEG expression (Egr family; Arc; ΔFosB) in different brain areas of mice and rats. In addition, the summative effects of later single/multiple exposure to stressful conditions in ELA subjects on IEG alteration are also investigated. Sex-dependent effects and other IEGs than c-Fos (Fos) are not investigated due to small number of publications. Although this manuscript addresses a really interesting topic and the results could be interesting for the readers of PlosOne, there are some concerns:

**Major:**
1. Abstract. I think that you have to describe the effects of ELA before the effects of ELA plus acute stress.

We thank the reviewer for the comment. However, after ample thought we have decided to leave the order as it was: This order makes it easier for the reader to understand our statement that the ELA phenotype seen under rest resembles that seen (in controls) after acute stress.

2. pg 17, line 282-284. Please rewrite this period, deleting the word “particularly”. Results indicate that cFos expression is increased in ELA animals at rest, while ELA plus acute stressed animals do not show a significant increase.

The word “particularly” has been removed from the sentence.

3. Fig 4. Please add the letters (A,B), clarify in the text the meaning of the words “single”, “multiple” and use the right terms. Please, also use the legend to explain better.

The figure’s labels have been changed with “no additional” and “additional” traumatic life events. Their meaning has been better clarified in the figure caption:

***“Fig 4. Main and subgroup analyses.*** *A) Effects of ELA on cFos expression in male rodents at rest and after an acute stressor. B) Subgroup analysis for absence (No Additional Hits) or presence (Additional Hits) of additional negative life events. Of note, control and experimental animals always differed only in the presence/absence of ELA. Therefore, in the ‘Additional Hits’ comparison, also control animal experienced the additional negative life events. * p < 0.05.”*

4. The Authors declare in the Method section to restrict the field of investigation focusing on early environmental manipulations sharing the alteration in maternal cares (lines 111-112). Because it is the main factor influencing the literature review/meta-analysis, this should be stressed when appropriate (e.g. title; abstract; introduction).

We agree with the reviewer and have included in the abstract and introduction the specification of ELA as “alteration of maternal care”.

5. The Authors affirm in Abstract that that they “meta-analyzed publications investigating specifically cFos expression”. Please, focus only on cFos expression (instead of on immediate early gene expression in general) throughout the manuscript. Change the title accordingly.

The manuscript focuses on the effects of ELA as alteration of maternal care on several immediate early gene’s expression. Due to the limited number of publications available, only cFos could be meta-analysed. Nevertheless, we provide a systematic review of all other outcomes (see S2.4). For this reason, we have not changed the title as suggested. We adapted the abstract, though, to describe exactly what we meta-analyzed.

6. I think that the direction (improve, impairment) of alteration in maternal cares reported in the selected papers should be considered as a critical element.

In S1.1, Item #13 in the table, all ELA models have been appropriately described and referenced. In general, ELA is always referred to as “impairment inducing” in induced models. The only non-induced ELA model included was “licking and grooming”. In this model, low licking and grooming is considered as “impairment inducing”. Models such as “handling for ~15min” of more unclear interpretation were excluded from the meta-analysis, as specified by the inclusion criteria.

7. The investigation of stress level (from none to severe) in this study is a precious contribution that Authors could deeply evaluate supporting its role in IEG expression and arranging comparisons throughout all manuscripts.

We agree with the reviewer that categorizing stress levels (from none to severe) is a useful (although not trivial) contribution to the field. However, we feel that our categorization mainly has exploratory value, as specified in **line 277-279**. Therefore, we are very careful in interpreting the related conclusion.

8. pg 21, line 346-349. I do not agree with the sentence “Increased activity…neutral condition”; you can’t conclude that the effects are related to “sensitization”. Replace the word “sensitization” with a more general term (i.e. alteration) and dampen the conclusions.

We have changed the word “sensitization” to “alteration” in **line 355**. In **line 356-359**, we have better specified that the suggestion of “synaptic sensitization” is a speculation.

*“Functionally, increased IEG expression at rest could reflect a differential, less adaptive way of processing previous experiences and could potentially hint towards an overall increased transcriptional activity as a result of synaptic alteration.”*

**Supplementary**
9. Please clarify why author choose to evaluate only on a systematic review level “stressors with a strong memory, social or reward component”.

As specified in the Supplementary Material **section 2.2.3** “*They involve cognitive processes other than the response to stress, which recruit brain-areas depending on the task requirements.*”. A reference to this sentence has now also been added to the inclusion/exclusion criteria section (**line 235**).

**Minor:**
Please check the consistency for the terms “single/multiple stress/hit/stressor” throughout all the manuscript and tables.

We thank the reviewer for the comment. We have changed the terminology throughout the text for consistency and clarity. In particular, we use “stressor” only in relation to “acute stress”, and we use “additional hits” for the experience of additional negative life events, in line with the multiple-hits theory.

Line 47-51
Different animal models of perturbation of early life environment fall under the definition of ELA and accordingly a plethora of studies report different long term effects of these depending on the procedure, strain, sex and other variables. I suggest dampening the sentence “ furthermore….and decrease social behavior” (pg 3, line 47-51) because this is only related to one paper from the same group.

New references have been added to support this claim.

Line 69
I suggest to choose between the words “several” and “different”. Use one of the two terms consistently.

These words have been critically assessed for correctness throughout the manuscript.

Line 113
Please clarify what “grooming and licking” model refer to. Are these selected from natural population variation, selected animal lines and/or genetic driven differences?

A detailed description of the model with the relevant references can be found in reference [10] (Liu et al., 1997). Briefly, the model refers to natural population variation as measured by observations (normally of a blinded observer). Selection of animal lines and specific genetic strains were excluded from the analysis, as specified in the exclusion criteria (see S1.1, Item # 24).

Line 214-216
please check the percentage values reported.

The percentage values sum to 100.01% due to rounding. Adding additional digits will render the figure less legible.

Line 334
please check which IEG results (cFos) the sentence refers to.

The sentence at **line 334** (now **347**) has been rephrased for better clarity:

*“Nonetheless, we observed a consistent, positive standardized mean difference in cFos expression after ELA across five out of the six quantitatively investigated brain regions.”*

Add in the table age of testing and time of the tissue collection following stress exposure. It could be useful and informative for readers.

Age of testing, as well as time between stress exposure and tissue collection is available in the full dataset. Of note, only adult animals (older than 8 weeks) were included in the analysis. Due to readability as well as space limitations in the tables, these factors (age/time between stress exposure and tissue collection) are not included in the tables of the publication, although they can be easily accessed in the online dataset (link to file: <https://osf.io/s2ae3/>).

Since stress is a relevant element assessed in present work, please arrange the reported publications in a table according to the categorization of acute stress- from none to mild to severe.

Although this is a good suggestion, there is a practical consideration that prevents such a choice. Thus, often the same publication reports on results of acute stress of different severity (e.g. none and mild). After ample consideration, we preferred maintaining comparisons from the same publications together.

The label “notes” in column is unclear.

The label “notes” has been changed to “Exp design details”.

Caption
Tab 1 _ I suggest to insert “position” specification at the end of “area acronyms”.

We agree. The position specification has been moved to the end of ‘area acronyms’.

Fig 3 _ Please check the legend (Fem= females ?).

Figure 3 caption has been clarified, and it is currently in the following format:

*“Fig 3. A) Study characteristics and B) Investigated brain areas reported as percentage of experiments. Fem = females.”*

**Reviewer #3:** Schuler et al have used metanalysis to determine changes in IEGs following ELA. This is an interesting study which is informative to the field. Whereas overall the study addresses a common topic is well designed and statistically appropriate, there are several concerns:

1. Early-life adverse experiences are lumped together: The type of early-life stress experienced can have vastly different outcomes on brain development and function, significantly altering behaviour later in life. While the authors describe that they performed sensitivity analysis for type of early-life adversity (ELA) models. This is insufficient, because the majority of studies used maternal separation. it would be interesting to have a better understanding of the ‘other’ group and how these results differed from the rest. If there is no way to properly account for this it would be useful to have some discussion of this in the text.

We agree with the reviewer that the type of ELA can have different outcomes on brain and behavior. Of the 20 studies ultimately included in the meta-analysis, only 2 did not use the maternal separation model (1 used the limited nesting and bedding model, and 1 used the licking and grooming model). The sensitivity analysis shows that these two publications do not have major (statistical) impact on the results; however, to better clarify the “other” group, we have added two sentences in text (**lines 290-293**) describing the results of these publications at the review level, rather than quantitatively.

*“Of the 20 publications included in the meta-analysis, only two did not use maternal separation as an ELA model [49, 59]. Despite the use of another model, the results of these studies align with the above findings, reporting no significant differences after acute stress in the areas meta-analytically investigated.”*

2. The included studies also varied in the use of stressors. The type of stressor can significantly alter patterns of cfos within the brain (Maras et al 2014), and there is excellent evidence for differential activation of for example, hippocampus by restraint vs footshock. Is it possible to adjust for type of stressor used if not done so already? Or further subdivide this analysis to include only those experiencing a comparable stress?

We thank the reviewer for the critical question. Although different types of acute stress can cause differential activation throughout the brain, in this meta-analysis we investigate the difference between controls and ELA animals, rather than the c-fos distribution within/between brain areas. We reasoned that the effects of ELA may interact with other factors for acute stressors with a strong memory, social or reward component. For this reason, we review these experiments only at a systematic review level (S2.4.3). However, for stressors with a physical component, we reasoned that the difference in effects between ELA and control should be comparable. In the 28 experiments using acute stressors, 10 different “types” of acute stressors were used. Due to the heterogeneity, it is not possible to quantify the different types with a subgroup analysis, although we did further categorize them (S1.4B) into mild/severe for subgroup analysis (results reported in **lines 287-290**). Additionally, the column ‘Exp design details’ in Table 1 specifies the stressor type of each experiment, such that interested reader can qualitatively assess the effects of a stressor of interest.

3. The combining of both mice and rats could be problematic. The authors state that they made the a priori decision to analyse females separately due to known differences. I think the same could be said about mice and rats as they have significant functional and anatomical differences. While much of this would be controlled for by using the within study effect sizes, it could be diluting effects of post-stress activation. Were any analyses for an effect of species performed? Or included as a co-variate?

The distribution of publications using rats or mice was not homogeneous, with rats being used in ~84% of publications. We analyzed together mice and rats (rather than focusing only on rats) to enhance the translational potential. For this particular outcome, we did not expect either of the two species to be more relevant for the human population. Although it is not possible to perform subgroup analysis on species –given the rather low number of publications in mice-, a sensitivity analysis has been performed on rats (S2.2). Briefly, the overall effect size was virtually unchanged, from hedge’s g (sem) = 0.223(0.075) of the complete model (rats and mice) to hedge’s g (sem) = 0.23(0.079) of the rat only model. The same pattern is found when analyzing only the experiments with an acute stressor: the effect size goes from hedge’s g (sem) = 0.109(0.11) of the full model to hedge’s g (sem) = 0.084(0.12) of the rat only model (S2.2). Although this does not mean that the effects are comparable for mice and rats, the conclusions drawn from the currently available evidence are robust to species. We have included the sensitivity analysis in the supplementary material (**S2.2**), as well as a remark in the manuscript (**line 201-203**):

“*Of note, since 76.3% of publications were in rats, the results are robust to rats, but more definite conclusions about mice (and potential inter-species differences) can only be drawn when more information is available on mice.”*

4. The authors mention some outliers. It would be helpful to inform the reader further about what is different about these studies. Could it be due to any of the above factors, do they use a different model of early life stress?

One comparison was excluded as an influential outlier, following a conservative statistical approach as described in reference [36]. The study used maternal separation in male rats and the comparison looked at cFos expression in the hypothalamus after restraint stress; the publication was not from a predatory journal. No element of the experimental design “stood out”, and we therefore interpreted this particular comparison as a statistical (rather than biological) outlier.

We have added the following in **line 178** to clarify the outlier removal procedure:

*“[Influential outliers were determined in accordance with Viechtbauer and Cheung [36] and removed from quantitative synthesis. Of such comparisons, we explored whether elements of the experimental design could explain the deviation of these comparisons from the mean.”*

In addition, we included qualitative information on the outlier in **line 232-234**:

*“No element of the experimental design pointed towards a biological origin of the outlying value, nor was its publication published in a predatory journal.”*

5. Some of these points could be addressed with the addition of a forest plot detailing the effect sizes for the studies included. Including some annotation or grouping of these differing factors could be informative.

In response to the reviewer, we have added a forest plot (S2.3) organized by species, ELA model, presence of additional traumas and type of acute stressor.
