# Supplementary material for: Effects of early life adversity on immediate early gene expression: Systematic review and 3-level meta-analysis of rodent studies
Source: PLoS One. 2022 Jan 13;17(1):e0253406. doi: 10.1371/journal.pone.0253406 (PMC8757918; doi:10.1371/journal.pone.0253406)
Supplement: Attachment — Submitted filename: mIEG_reviewerscomments_rebuttal_02.docx [file pone.0253406.s004.docx]

**PONE-D-21-18961R1**

**Title**: Effects of early life adversity on immediate early gene expression: systematic review and 3-level meta-analysis of rodent studies

Schuler & Bonapersona, et al.

**Answers to reviewers’ comments**

Of note, in the Editor’s letter, we were requested to address only the points of Reviewer #3.

**Reviewer #3:**

 The authors addressed most of the concerns in a reasonable manner. A few additions will be helpful:

1) (Referring to original comment 2) It would be useful to include the cross-reference to the definition in the supplement of mild/severe in the relevant results section. I couldn’t find the relevant report on lines 287-290 and assume this is an error and the authors are referring to the analysis on lines 308-312 and think this information should be included here. The authors reasoning regarding the choice of stressors included in the analyses (as detailed above) and the heterogeneity should be included in the correct section.

**Answer:** We apologies for wrongly referencing the requested analysis in the rebuttal letter. The results in question are described in **line 286-288** of the main manuscript.

We report on all exploratory analyses in lines 284-295. The analysis on the moderator effects of stressor severity was part of the exploratory analysis. Therefore, we believe that this section is the appropriate one to report its results.

We agree with the reviewer that the definition of stressor severity could be more clear. Although this was already defined in the Methods section (see lines 190-192), we have added an extra sentence in the results section to improve clarity. As present in lines 285-286, it now reads:

“*For the categorization of acute stressor severity, please see the Supplementary Section S1.4.”*

Additionally, we have edited the Supplementary Section S1.4. Supplementary section S1.4 now reads:

‘S*tressors with a strong memory, social or reward component were assessed on a systematic review level only. Although different types of acute stress can cause differential activation throughout the brain, in this meta-analysis we investigate the difference between controls and ELA animals, rather than the c-fos distribution within/between brain areas. The effects of ELA may interact with other factors for acute stressors with a strong memory, social or reward component. For this reason, we review these experiments only at a systematic review level (S2.4.3). However, for stressors with a physical component, we reasoned that the difference in effects between ELA and control should be comparable.”*

In addition, in the 28 experiments using acute stressors, 10 different “types” of acute stressors were used. Due to the heterogeneity, it is not possible to quantify the different types with a subgroup analysis, although we did further categorize them into mild/severe for subgroup analysis (results reported in lines 286-288). Additionally, the column ‘Exp design details’ in Table 1 specifies the stressor type of each experiment, such that interested reader can qualitatively assess the effects of a stressor of interest.

Overall, we believe that these additions fully answer the reviewer’s comment.

2 ) (Referring to original comment number 4 of reviewer 3 of first round of reviews) Thanks for the added information, it may be useful to include information regarding the definition of predatory journal that was used eg. <https://www.nature.com/articles/d41586-019-03759-y>

In this case, we confirmed that the excluded observation was reported in journals by well-known, reputable publishers (Journal of Neuroendocrinology, Wiley). Furthermore, only one comparison was excluded from this publication, whereas no other comparisons were considered influential outliers. Nevertheless, we have added the reference suggested by the reviewer to the manuscript (line 231, reference [75]).
